# Cancer Radiation Therapy May Be Associated With Atrial Fibrillation

**DOI:** 10.3389/fcvm.2021.610915

**Published:** 2021-01-22

**Authors:** Nachiket Apte, Parinita Dherange, Usman Mustafa, Lina Ya'qoub, Desiree Dawson, Kathleen Higginbotham, Marjan Boerma, Daniel P. Morin, Dipti Gupta, Jerry McLarty, Richard Mansour, Paari Dominic

**Affiliations:** ^1^Department of Medicine and Center of Excellence for Cardiovascular Diseases & Sciences, Louisiana State University Health Sciences Center-Shreveport, Shreveport, LA, United States; ^2^Ochsner-LSU Medical Center, Shreveport, LA, United States; ^3^Department of Pharmaceutical Sciences, University of Arkansas for Medical Sciences, Little Rock, AR, United States; ^4^Department of Cardiology, Ochsner Medical Center, New Orleans, LA, United States; ^5^Department of Cardiology, University of Queensland Ochsner Clinical School, New Orleans, LA, United States; ^6^Cardiology Service, Department of Medicine, Memorial Sloan Kettering Cancer Center, New York, NY, United States; ^7^Department of Medicine and Feist Weiller Cancer Center, Louisiana State University Health Sciences Center-Shreveport, Shreveport, LA, United States

**Keywords:** atrial fibrillation, cancer, radiation therapy, chemotherapy, solid malignancy, hematological malignancies

## Abstract

**Background:** The association of atrial fibrillation (AF) with cancer and cancer types is inconclusive. Similarly, data regarding the association of AF with different cancer therapies are controversial.

**Objectives:** To study the association of AF with cancer subtypes and cancer therapies.

**Methods:** We studied all patients aged 18–89 years who presented to the Feist Weiller Cancer Center, with or without a diagnosis of cancer, between January 2011 and February 2016. Electronic health records were systematically queried for baseline demographics and ICD-9 and ICD-10 codes for specific co-morbidities. Patients with a diagnosis of AF were tabulated based on cross-validation with the ECG database and/or by recorded history. We assessed the prevalence and risk of AF based on cancer diagnosis, specific cancer type, and cancer therapy.

**Results:** A total of 14,600 patients were analyzed. Compared to non-cancer patients (*n* = 6,801), cancer patients (*n* = 7,799) had a significantly higher prevalence of AF (4.3 vs. 3.1%; *p* < 0.001). However, following correction for covariates in a multivariable logistic regression model, malignancy was not found to be an independent risk factor for AF (*p* = 0.32). While patients with solid tumors had a numerically higher prevalence of AF than those with hematological malignancies (4.3 vs. 4.1%), tumor type was not independently associated with AF (*p* = 0.13). AF prevalence was higher in patients receiving chemotherapy (4.1%), radiation therapy (5.1%), or both (6.9%) when compared to patients not receiving any therapy (3.6%, *p* = 0.01). On multivariable logistic regression, radiation therapy remained an independent risk factor for AF for the entire study population (*p* = 0.03) as well as for the cancer population (*p* < 0.01).

**Conclusions:** Radiation therapy for cancer is an independent risk factor for AF. The known association between cancer and AF may be mediated, at least in part, by the effects of radiation therapy.

## Introduction

The term “cardio-oncology” describes a growing medical specialty working at the intersection of cardiovascular diseases and cancer. Among the cardiotoxic effects of cancer and cancer therapy, cardiomyopathy and coronary artery disease have been well-studied, but the relationship between cancer and cardiac arrhythmias has been examined less. Most of the studies evaluating the association between atrial fibrillation (AF) and cancer have been limited to the postoperative period, or specifically involve the use of certain chemotherapeutic agents with known cardiotoxicity, such as ibrutinib and doxorubicin. Based on a population based study by Hu et al. (1), 2.4% of patients with cancer had AF at the time of cancer diagnosis, and 1.8% developed new-onset AF after the diagnosis of cancer. Cancer and AF may share risk factors or pathophysiological processes (2, 3) and there is some evidence that AF may be a marker of underlying cancer (4). The proposed mechanisms for the pathogenesis of AF in cancer patients, other than common risk factors such as advanced age, obesity, diabetes, and smoking, include cancer-related conditions such as increased inflammation, fibrosis, and hypercoagulability-induced pulmonary micro-embolism, all of which trigger AF (5). While there is evidence for the occurrence of AF following surgery for colorectal and breast cancer (5–7), it remains unclear whether cancer itself is independently associated with the risk of AF, or whether the prevalence of AF is increased in patients with cancer due to coexistent co-morbidities.

We assessed the prevalence of AF in patients with various types of cancer and the association of AF with cancer therapy, including chemotherapy and radiation therapy.

## Methods

### Patient Selection and Data Collection

Approval was obtained from the Institutional Review Board at Louisiana State University Health Sciences Center (LSUHSC) at Shreveport for this study. All patients between the ages of 18 and 89 years who presented to LSUHSC and the Feist Weiller Cancer Center between January 2011 and February 2016 with a diagnosis of cancer were included in the study. They were compared to a control population who presented to the cancer center without a diagnosis of cancer, most of whom were patients with non-malignant hematological conditions.

**Figure 1:**
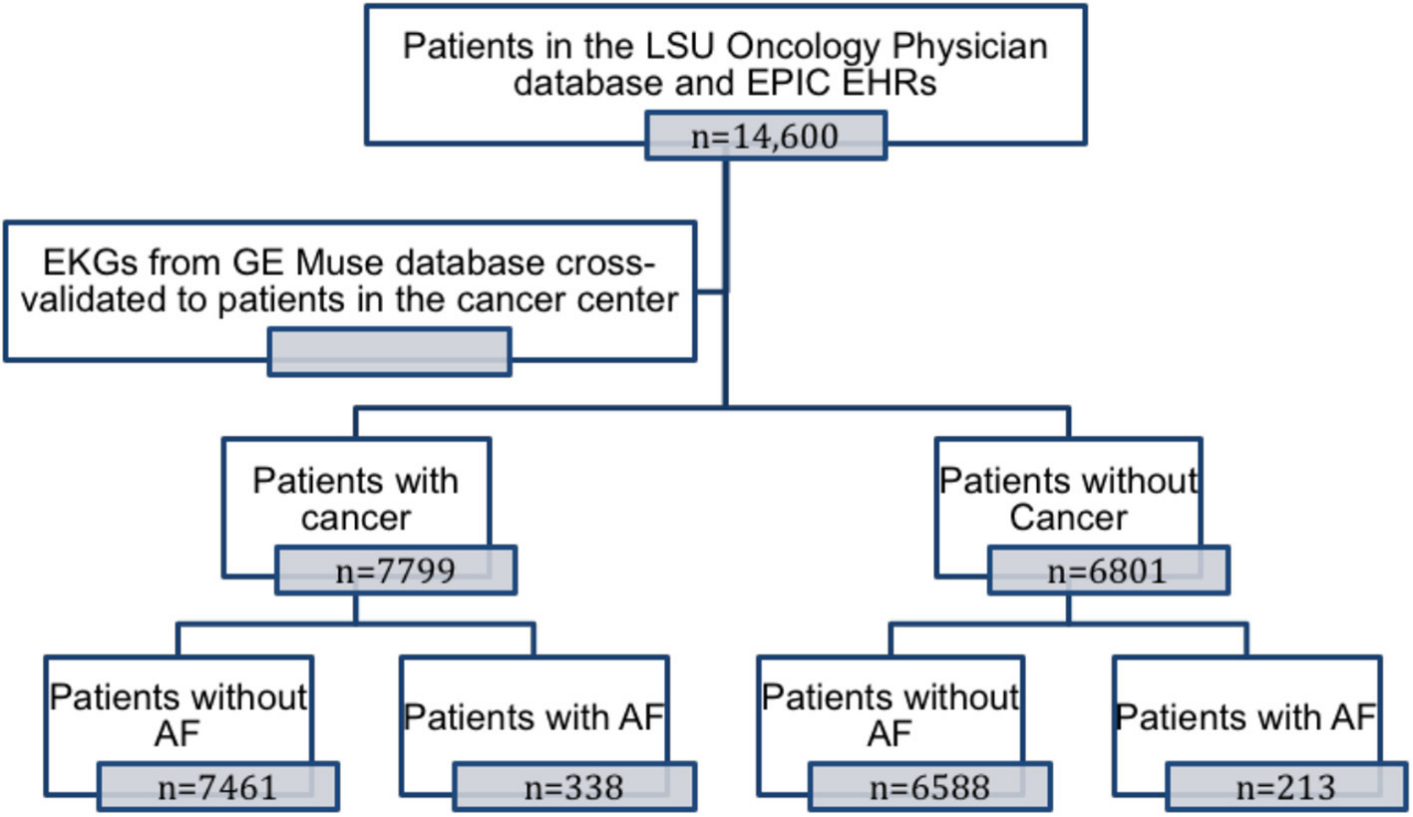
Patients included in the study.

Patient enrollment in the study is shown in Figure 1. Patients were categorized as having cancer if they had a solid or hematological malignancy. Electronic health records from both the hospital system (EPIC Electronic Health Record System) and the Cancer Center System (Sunrise Electronic Health Record System) were systematically queried for ICD-9 and ICD-10 codes to assess baseline clinical characteristics and co-morbidities (list of ICD-9 and ICD-10 codes available in the Supplementary Material). Medication history, including cancer therapy regimens and procedures like radiation therapy, were obtained from the EMR and from the local tumor registry. For each patient, all electrocardiograms (ECG) in the MUSE^TM^ (GE) electronic ECG reading system were systematically queried for AF. A patient was considered to have AF if he/she had a documented history of AF in the electronic health record system at the hospital or the cancer center, and/or had an ECG diagnosis as confirmed by a cardiologist in the MUSE^TM^ system. The age of the patient was calculated using the date of the last office visit and his or her birth date.

### Statistical Methods

Statistical analysis was performed using IBM SPSS Statistics (version 26.0; IBM Corporation, Armonk, NY). Continuous data are expressed as the mean ± standard deviation (SD) or median and interquartile range, and categorical variables are expressed as absolute numbers and percentages. Independent-sample *t*-tests were used to compare continuous data between groups. The Kruskal Wallis ANOVA test was used to evaluate differences between more than two independent groups. A number of exploratory analyses (including AF risk based on type of cancer, location of cancer, cancer chemotherapy used, and radiation therapy) were used in this study. For the most part, the Pearson's chi-square test (or Fisher's exact) was used for categorical variables. Multivariate logistic regression was used to compute adjusted odds ratios (OR) for binary outcome variables. Statistical significance was defined as two-sided *p* < 0.05.

## Results

### Prevalence of Atrial Fibrillation in Cancer Patients

**Figure 2:**
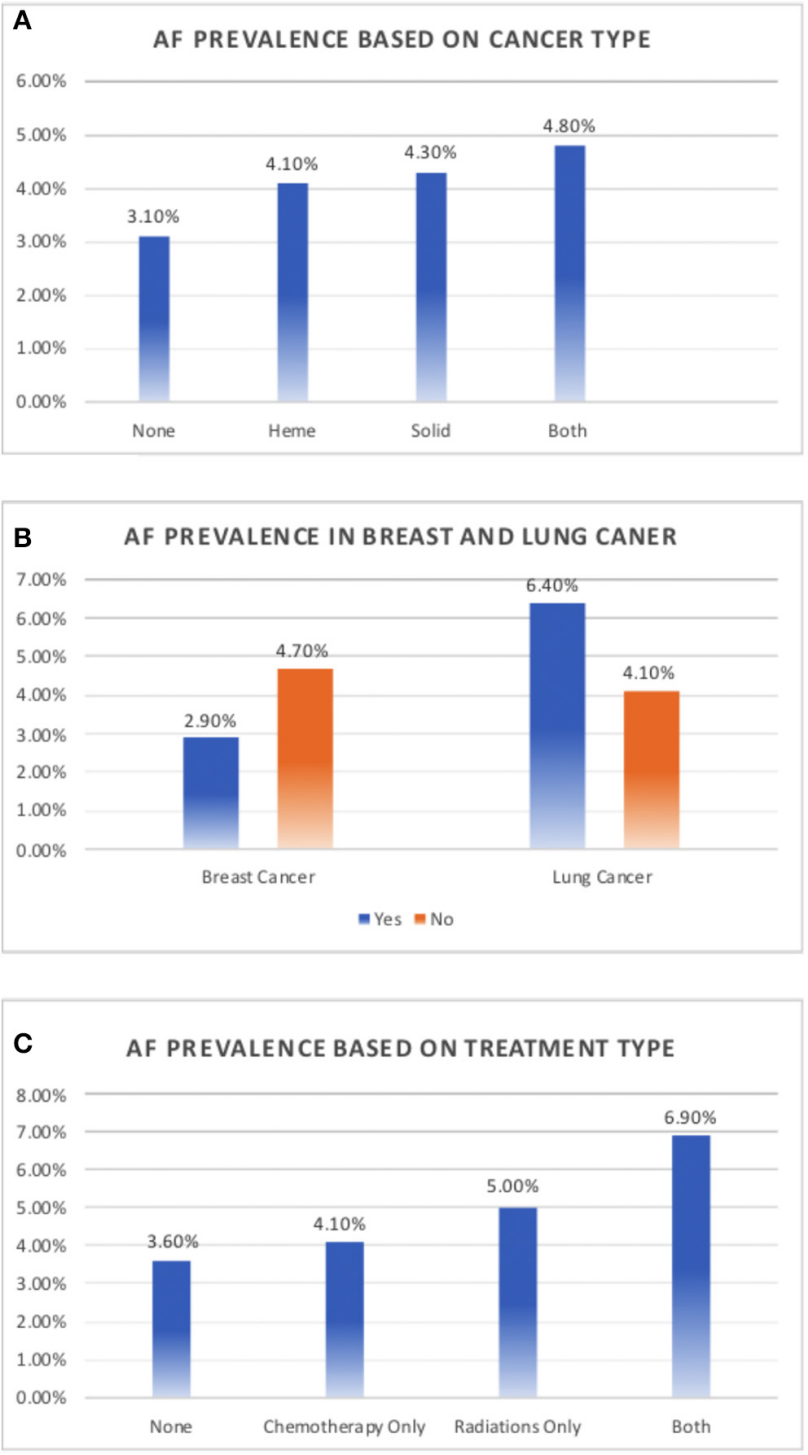
Prevalence of atrial fibrillation in cancer. **(A)** Prevalence of atrial fibrillation based on a history of solid tumor, hematological malignancy, or both, compared to non-cancer patients. **(B)** Prevalence of atrial fibrillation in patients with and without breast and lung cancer. **(C)** Prevalence of atrial fibrillation based on cancer therapy.

A total of 14,600 patients between the ages of 18 and 89 years presented to the Feist Weiller Cancer Center between January 2011 and February 2016. These included 7,799 patients with hematological or solid malignancies or both, and 6,801 patients without cancer (see Table 1 for characteristics). The majority of the cancer patients (72%) had a history of solid malignancies, 18% had hematological malignancies, and 10% had both. AF was prevalent in 551 (3.8%) of the total population. Among the cancer patients, 4.3% (338 patients) had a diagnosis of AF compared to 3.1% (213 patients) in the non-cancer population. As shown in Figure 2A, compared to non-cancer patients, the prevalence of AF was higher (*p* = 0.002) in patients with hematological malignancies (4.1%), solid malignancies (4.3%), or both (4.8%).

**Table 1:** Baseline characteristics and co-morbidities of patients with and without a diagnosis of cancer.

**Results**	**Patients with a diagnosis of cancer** **(*n* = 7,799)**	**Patients without a diagnosis of cancer** **(*n* = 6,801)**	***P*-value (2-sided)**
Age	60.3	52.6	<0.001
Race: African American White Others	53.1% 49.5% 2.1%	48.4% 43.6% 3.3 %	<0.001^*^
Sex: male	39.6%	33.0%	<0.001
BMI	28.2	30.1	<0.001
Tobacco use	62.7	37.3	<0.001
Years of smoking	28.2	23.2	<0.001
CAD	5.2%	3.8%	<0.001
Myocardial infarction	2.8%	2.8%	1.0
CHF	0.9%	0.8%	0.86
Atrial fibrillation	4.3%	3.1%	<0.001
Hypertension	42.6%	30.6%	<0.001
TIA/Ischemic stroke	3.8%	3.0%	0.01
Intra cranial hemorrhage	0.9%	1.0%	0.609
Diabetes mellitus	17.1%	13.5%	<0.001
DVT/PE	5.3%	3.3%	<0.001
OSA	0.4%	0.4%	1.000
CKD	2.7%	2.5%	0.47
ESRD	0.9%	1.5%	0.002
COPD	0.9%	0.5%	0.01

CAD, coronary artery disease; CHF, congestive heart failure; DVT/PE, deep venous thrombosis/pulmonary embolism; OSA, obstructive sleep apnea; CKD, chronic kidney disease; ESRD, end stage renal disease; COPD, chronic obstructive pulmonary disease.

* *Pearson's chi-square test*.

The characteristics of cancer patients with and without AF are tabulated in Table 2. Because the traditional AF risk factors (hypertension, age, diabetes, body mass index, and male sex) were more prevalent in cancer patients with AF, possibly explaining the higher prevalence of AF in our cancer population, a multivariate logistic regression model including these risk factors and cancer diagnosis was used to assess whether cancer was an independent risk factor for AF. While traditional risk factors remained associated with the prevalence of AF, with the highest risk associated with age and hypertension, malignancy was not found to be an independent risk factor for AF (*p* = 0.32). Interestingly, while diabetes and tobacco use were associated with cancer risk in the general population (OR 1.38, 95% CI 1.11–1.71, *p* = 0.004 and OR 1.27, 95% CI 1.04–1.54, *p* = 0.02, respectively), as shown in **Table 5**, when studied in patients with cancer, these two risk determinants were not statistically significantly associated with AF risk.

**Table 2:** Baseline characteristics and co-morbidities of cancer patients with and without a diagnosis of atrial fibrillation.

**Results**	**Cancer patients with atrial fibrillation** **(*n* = 338)**	**Cancer patients without atrial fibrillation** **(*n* = 7,461)**	***P*-value (2-sided)**
Race: African American White Others	45.3% 52.7%	48.5% 49.3%	0.489^*^
Sex: Male	55.0%	38.9%	<0.001
CAD	18.0%	4.6%	<0.001
Type: Solid Hematological Both	72.2% 16.9% 10.9%	72.3% 17.9% 9.8%	0.74^*^
Myocardial infarction	10.0%	2.4%	<0.001
CHF	5.0%	0.7%	<0.001
Hypertension	57.7%	42%	<0.001
TIA/ischemic stroke	7.1%	3.7%	0.003
Intra cranial hemorrhage	1.8%	0.9%	0.13
Diabetes mellitus	25.7%	16.8%	<0.001
DVT/PE	10.7%	5.1%	<0.001
OSA	0.9%	0.4%	0.18
CKD	5.0%	2.6%	0.02
ESRD	3.0%	0.8%	0.001
COPD	2.7%	0.8%	0.003

CAD, coronary artery disease; CHF, congestive heart failure; DVT/PE, deep venous thrombosis/pulmonary embolism; OSA, obstructive sleep apnea; CKD, chronic kidney disease; ESRD, end stage renal disease; COPD, chronic obstructive pulmonary disease.

* *Pearson's chi-square test*.

### Type of Cancer and Atrial Fibrillation

While there was an increased prevalence of AF in patients with solid tumors (4.3%) compared to those with hematological tumors (4.1%; Figure 2A), the type of tumor was not independently associated with a prevalence of AF (*p* = 0.13) in the multivariate logistic regression model.

We evaluated the association between each cancer type and AF risk in the total study population using unadjusted chi-square test and adjusted logistic regression model (Table 3). Breast cancer contributed the largest population of patients with a history of malignancies, representing 24% of all cancer patients (*n* = 1,872). Interestingly, among patients with a history of cancer, breast cancer patients had a lower prevalence of AF, with only 2.9% having a concomitant diagnosis of AF compared to 4.7% of patients with other malignancies (*p* = 0.001; Figure 2B). On further analysis, this decreased prevalence was explained by the fact that most patients with breast cancer were female, which is consistent with documented evidence that the female sex is associated with a decreased prevalence of AF (6, 7). There were 9,267 females in the study, of whom 2.8% had history of AF, compared to 5.6% of the 5,333 males (*p* = 0.001). When adjusted for sex, there was no difference in the prevalence of AF in breast cancer patients compared to patients with other malignancies (*p* = 0.66). But when adjusted for traditional risk factors of AF, including sex, in the entire study population, breast cancer was independently associated with decreased risk of AF (OR-0.65; 95% CI-0.47–0.89, Table 3)

**Table 3:** Unadjusted prevalence of AF and adjusted odds ratio of AF risk based on cancer types.

**Cancer type**	**No. of pts with cancer**	**No. of pts without cancer**	**AF pts with cancer type (%)**	**AF pts without cancer type (%)**	**Unadjusted *p*-value**	**Adjusted OR, 95% CI (lower-upper)**	**Adjusted *p*-value**
Lung	873	13,727	6.4	3.6	<0.01	1.14 (0.82–1.58)	0.44
Prostate	239	5,094	8.8	5.4	0.025	0.92 (0.56–1.51)	0.75
Breast	1,872	12,728	2.9	3.9	0.02	0.65 (0.47–0.89)	0.01^*^
Colorectal	675	13,925	4.6	3.7	0.25	0.88 (0.59–1.29)	0.51
GU	1,223	13377	4	3.8	0.66	0.89 (0.65–1.22)	0.48
Thyroid	111	14,489	7.2	3.8	0.06	2.01 (0.95–4.27)	0.07
Upper GI	746	13,854	5.9	3.7	<0.01	1.5 (1.08–2.09)	0.02^#^
Head & Neck	1,081	13,519	4.4	3.7	0.30	0.81(0.59–1.12)	0.20
Neurologic	420	14,180	3.1	3.8	0.46	0.82 (0.46–1.45)	0.49

GU, genitourinary; GI, gastrointestinal; AF, atrial fibrillation; OR, odds ratio; CI, confidence interval. Only males were included in the evaluation of prostate cancer associations; skin, eye, other endocrine malignancies were not analyzed.

* P-value signifies a protective effect of breast cancer on AF risk.

# *P-value signifies an association of increased risk of AF in upper GI cancers. GU cancers excludes prostate cancers; unadjusted p-value based on chi-square test and adjusted OR and p-value based on logistic regression*.

We also assessed the prevalence of AF in the 873 lung cancer patients in the study. The prevalence of AF was significantly higher in lung cancer patients than in patients with other malignancies (6.4 vs. 4.1%, *p* < 0.01; Figure 2B). In the total study population, 6.4% of patients with lung cancer had AF compared to 3.6% of patients without lung cancer (*p* < 0.01) but when adjusted for traditional risk factors of AF, lung cancer was not associated with increased risk of AF (OR-1.14; 95% CI- 0.82–1.58, *p* = 0.44, Table 3).

Among all the cancer types evaluated, upper GI cancers (*n* = 746) were associated with a higher risk of AF based on the unadjusted chi-square test (5.9% AF in patients with upper GI cancer compared to 3.7% in patients without, *p* < 0.01) and the adjusted logistic regression model (OR-1.5; 95% CI- 1.08–2.09, *p* = 0.02, Table 3).

### Cancer Therapy and Atrial Fibrillation

Among all patients included in the study, patients undergoing chemotherapy or radiation had an increased prevalence of AF. Compared to patients who received neither chemotherapy nor radiation (*n* = 12,307, AF-3.6%), patients who underwent chemotherapy (*n* = 1,665) had a 4.1% prevalence of AF. The prevalence of AF further increased to 5.1% in patients who underwent radiation therapy (*n* = 322) and 6.9% in patients who were subjected to both (*n* = 306, *p* = 0.013; Figure 2C). A multivariable logistic regression model including the traditional AF risk factors showed that a history of chemotherapy was not independently associated with AF risk (*p* = 0.23). In addition, the number of chemotherapeutic drugs was not associated with AF in cancer patients (*p* = 0.59). The prevalence of AF was 4.3% in patients who received no chemotherapeutic drugs (*n* = 5,895), 5.8% in patients who received one drug (*n* = 637), 4.1% in patients who received two drugs (*n* = 731), 3.6% in patients who received three drugs (*n* = 384), and 3.1% in patients who received four drugs (*n* = 130). In addition, we assessed AF prevalence associated with individual chemotherapeutic agents (Table 4). Of the drugs analyzed (cyclophosphamide, paclitaxel, docetaxel, trastuzumab, carboplatin, cisplatin, idarubicin, mitoxanthrone, and doxorubicin), only idarubicin and mitoxanthrone were associated with a significantly higher prevalence of AF. The number of patients receiving these two chemotherapeutic agents was very small, precluding a valid multivariable regression.

**Table 4:** Risk of AF based on exposure to chemotherapeutic agents.

**Chemo drug**	**No. of pts on drug**	**No. of Pts not on drug**	**AF in pts exposed to drug (%)**	**AF in pts not exposed to drug (%)**	***p* Value**
Cisplatin	374	7,425	4.8	4.3	0.64
Cyclophosphamide	780	7,019	3.7	4.4	0.37
Docetaxel	499	7,300	3.4	4.4	0.29
Doxorubicin	602	7,197	3.2	4.4	0.14
Etoposide	71	7,728	11.3	4.3	0.01
Gemfibrozil	206	7,593	3.9	4.3	0.75
Idarubicin	21	7,778	19	4.3	0.01
Mitoxantrone	7	7,792	28.6	4.3	0.03
Paclitaxel	770	7,029	4	4.4	0.66
Trastuzumab	154	7,645	1.9	4.4	0.14

*AF, atrial fibrillation. Only cancer patients were included in this analysis; chi-square test was used unless cell values <10 in which case Fisher Exact test used*.

Among the patients included in the study, 628 patients (4.3% of all patients and 8.1% of all cancer patients) received radiation therapy. When considering all patients in the study, patients who received radiation therapy had a significantly higher prevalence of AF compared to patients who did not (5.9 vs. 3.7%; *p* < 0.01). Similarly, when considering only patients with cancer, patients who received radiation therapy had a significantly higher prevalence of AF compared to patients who did not receive radiation therapy (5.9 vs. 4.2%; *p* = 0.046). As shown in Table 5, a multivariate logistic regression model with traditional risk factors of AF showed that radiation therapy remained an independent risk factor for AF whether considering the entire study population (*p* = 0.03) or only patients with cancer (*p* < 0.01).

**Table 5:** Adjusted odds ratios for the risk factors of AF in cancer patients and controls.

**Adjusted odds ratios for the risk of AF**						
	**All patients**			**Cancer patients**		
	**Adjusted OR**	**95% confidence interval**	***P*****-value**	**Adjusted OR**	**95% confidence interval**	***P*****-value**
Age	1.06	1.05–1.07	<0.001	1.07	1.06–1.08	<0.001
HTN	1.53	1.26–1.85	<0.001	1.52	1.20–1.93	<0.001
DM	1.38	1.11–1.71	0.004	-	-	0.09
Tobacco	1.27	1.04–1.54	0.018	-	-	0.313
BMI	1.02	1.01–1.03	0.002	1.02	1.004–1.03	0.01
Race	-	-	0.055	-	-	0.073
Cardiomyopathy	7.11	5.34–9.48	<0.001	6.85	4.76–9.86	<0.001
COPD	2.79	1.42–5.48	0.003	3.25	1.51–6.99	0.003
Gender (Male)	2.06	1.71–2.49	<0.001	2.22	1.75–2.82	<0.001
Chemotherapy	-	-	0.23	-	-	0.154
Radiation therapy	1.49	1.04–2.14	0.028	1.63	1.13–2.36	0.009

*HTN, hypertension; DM, diabetes mellitus; BMI, body mass index; OR, odds ratio*.

### Sensitivity Analysis

Atrial fibrillation prevalence was identified in 551 patients in the total study population based on ECG diagnosis and/or ICD-9 and ICD-10 codes, of which 327 had ECG evidence of AF. A sensitivity analysis was performed for univariate association between cancer and AF using ECG proven AF diagnosis alone. AF prevalence significantly increased from 1.8% in non-cancer patients to 2.6% in cancer patients (*p* = 0.001). An increase of 44% more cases of AF in the cancer patients compared to controls when taking ECG proven AF alone was very similar to the 39% more cases of AF in the cancer patients compared to controls when taking ECG proven and or ICD-9 and ICD-10 based AF diagnosis, suggesting that the sampling of AF based on ICD-9 and ICD-10 codes were closely aligned with ECG based diagnosis. Similarly, AF diagnosis with or without an ECG evidence did not affect the results of the univariate AF association with radiation (Based on ECG and/or ICD codes- 5.9% of patients who underwent radiation had AF compared to 3.7% of patients who did not undergo radiation, *p* = 0.004; based on ECG alone- 4% of patients who underwent radiation had AF compared to 2.2% of patients who did not undergo radiation, *p* = 0.003).

## Discussion

Our study has several important findings. AF is more prevalent in patients with malignancies and in those who undergo chemotherapy, radiation therapy, or both. However, following multivariable analysis, only radiation therapy remained independently associated with AF. Thus, the association between cancer and atrial fibrillation may be mediated in part by an increased risk of AF among patients receiving radiation treatment.

### Prevalence of Atrial Fibrillation in Cancer Patients, Time Course and Mechanisms

**Figure 3:**
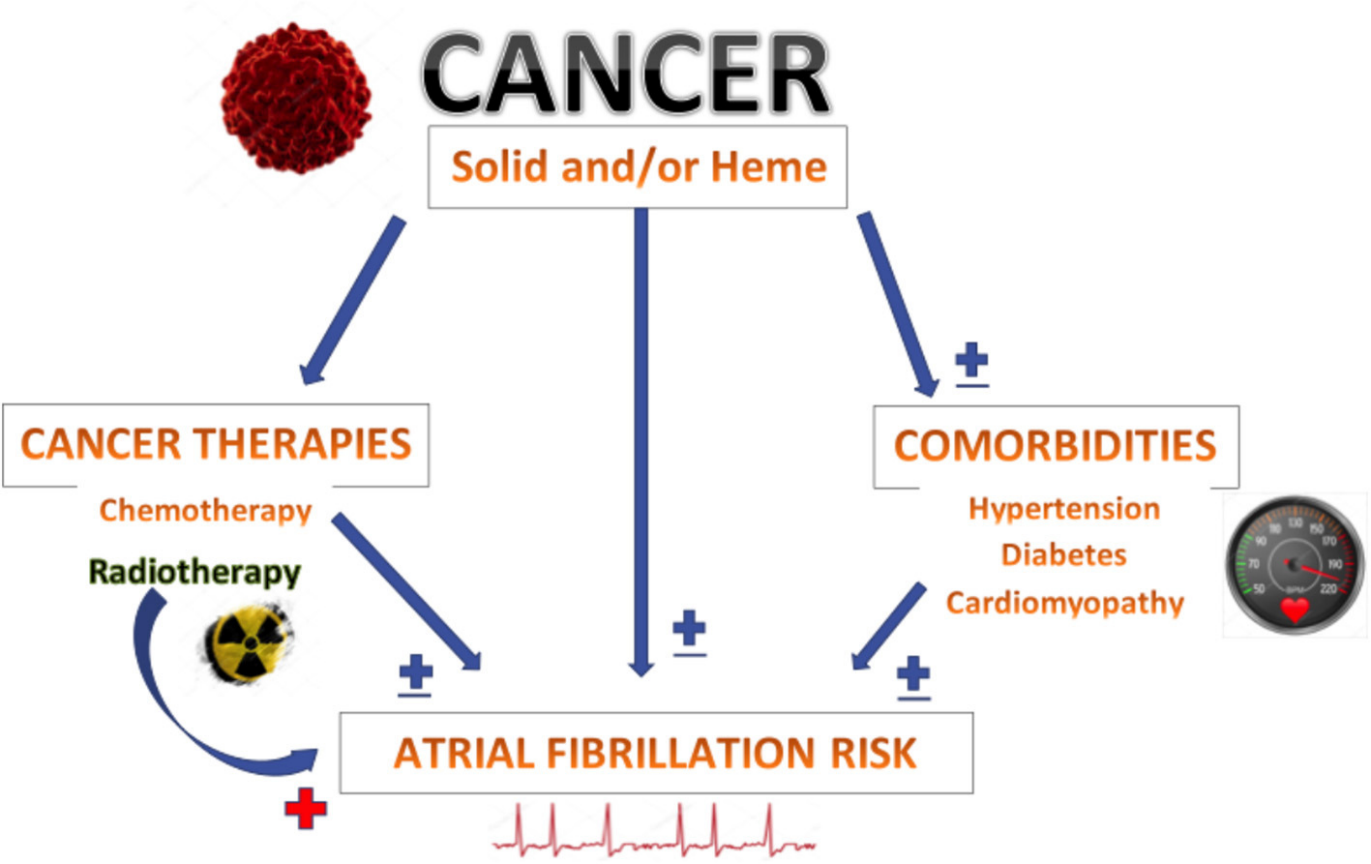
Both solid and hematological malignancies are associated with increased prevalence of atrial fibrillation. Based on our study, most of this increased risk appears to stem from associated comorbidities and when adjusted for these conditions, cancer by itself was not associated with increased risk of atrial fibrillation. While cancer chemotherapy and radiation therapy are also linked to increased prevalence of atrial fibrillation, only radiation therapy, regardless of the location, was independently associated with atrial fibrillation risk.

Atrial fibrillation, the most common cardiac arrhythmia, is frequently seen in patients with cancer. While elderly patients have a higher incidence of both AF and cancer, other co-morbidities in cancer patients may also predispose them to develop AF (8). Therefore, a higher prevalence of AF in cancer patients is to be expected. Some studies that have shown cancer to be an independent risk factor for AF did not adjust for traditional risk determinants of AF (9, 10) or found an increased risk as a result of detection bias in the immediate period following cancer diagnoses (11). While prior studies have shown a higher prevalence of AF in cancer patients similar to that demonstrated in our study, we find that when adjusted for co-morbidities, cancer itself does not appear to be an independent risk factor for AF (Figure 3). Our findings are similar to those in the study performed by Saliba et al. (12), which used a cohort study design to assess the risk of atrial fibrillation in ~20,000 patients within two large population-based case control studies consisting of patients with breast and colorectal cancers. Our study is also the first to show that radiation therapy for cancer, regardless of the location, independent of the co-morbidities found in cancer patients, is associated with a higher risk of AF (Figure 3).

Cancer is associated with a heightened risk for various forms of cardiovascular disease, including both fatal and benign arrhythmias (13, 14). Studies have shown that AF is more prevalent in cancer patients compared to non-cancer patients (1, 9, 11, 15), even when the cancer was non-life-threatening and the patients were not actively receiving cancer treatment (5). The incidence of AF has been shown to decline with time from the cancer diagnosis (16). Moreover, there is evidence that AF has been associated with worse outcomes and survival rates in these patients (17, 18), making the diagnosis of AF in cancer patients increasingly important. Conversely a study from a Danish registry suggested that AF could be a marker for occult cancer (19). In their study, patients with AF had a 2.5% risk of a cancer diagnosis in the first 3 months following AF diagnosis, a finding that could be confounded by detection bias. There are several hypotheses that could explain the higher prevalence of AF in cancer patients as it relates to the cancer itself or to the cancer treatment. These hypotheses include inflammation, which plays a key role both in carcinogenesis and AF (20, 21), cardiotoxicity caused by chemotherapy (22, 23), surgical treatment leading to postoperative AF, and autonomic dysfunction in cancer patients that may lead to increased sympathetic nervous system function (24, 25).

### Atrial Fibrillation and Type of Cancer

The evidence for the association of AF with any specific type of cancer is inconclusive. In our study, we found a significantly higher prevalence (*p* < 0.01) of AF in patients with hematological malignancies (4.1%), solid malignancies (4.3%), and both (4.8%) compared to non-cancer patients (3.1%), but neither of these tumor types were independently associated with a prevalence of AF. Whereas a case control study found a higher risk of AF in colorectal cancer patients, regardless of stage compared to controls, diagnosed at fewer than 90 days from the cancer diagnoses (11), we did not find such an association in our patient population. While a retrospective study by D'Souza et al. suggested a higher incidence of AF in female patients with breast cancer (26), we found no difference in the prevalence of AF in breast cancer patients in a separate gender-based analysis. Our results are similar to those from a large Danish cohort study by Vinter et al. who found no association between breast cancer and AF (4). Cancers involving the mediastinal and thoracic structures, such as the lung (16, 19), esophagus (16, 19), and mediastinal lymph nodes (19) are strongly associated with AF, possibly due to the local inflammatory milieu surrounding the heart. Our results show a higher prevalence of AF in patients with lung cancer, and upper GI cancers, consistent with these results. In addition, cancers that are associated with a high systemic inflammatory response, such as lung and colorectal cancers (27), seem to have a stronger association with AF.

### Atrial Fibrillation and Cancer Therapies

Although studies demonstrate that cancer patients have a higher risk of AF even before undergoing chemotherapy or radiation therapy (5, 10), multiple studies have shown an increased risk of AF during or following chemotherapy, possibly by causing structural and/or electrical remodeling, inducing and maintaining inflammation, and/or causing cardiac damage and cellular apoptosis (19, 21, 23). Certain chemotherapeutic agents have been associated with AF more than others, including tyrosine kinase inhibitors, anthracyclines, alkylating agents, epidermal growth factor inhibitors, and angiogenesis inhibitors (25). In our study, the anthracyclines idarubicin and mitoxanthrone were both associated with an increased risk of AF. The rest of the chemotherapeutic agents studied were not associated with an increased risk of AF in our patient population. It is noteworthy that ibrutinib, a novel tyrosine kinase inhibitor, is notoriously associated with AF, with an incidence reported to range between 5 and 16% of patients, but this agent was not analyzed in our study (23, 28). Finally, in addition to cancer chemotherapy, supportive medications used in cancer patients could also predispose patients to AF (29, 30).

Data concerning the impact of radiation therapy on the incidence of arrhythmias in cancer patients are scarce. Studies have shown that radiation therapy has been associated with the later development of coronary artery disease (CAD) (31, 32), heart failure (33) and conduction abnormalities (34). Specifically, radiation therapy to the left chest is associated with an increased risk of cardiovascular effects compared to radiation therapy to the right chest (25). Unfortunately, due to the small numbers of patients with radiation therapy in our study (628 patients, 4.3% of the total population), we could not further assess the relationship between laterality of radiation therapy and AF. Radiation therapy can cause myocardial fibrosis and autonomic dysfunction, leading to arrhythmias and elevated heart rate (24, 25).

## Limitations

Our study has several limitations. (1) This is a retrospective analysis of patients from a single center, so results may not be generalized to other populations. (2) Due to limitations in accurately documenting the time of diagnosis of AF and cancer, we opted to evaluate prevalence as opposed to AF incidence and therefore a time to event analysis was not performed. (3) Our study only included classical chemotherapeutic drugs and Trastuzumab and did not include drugs like Tyrosine kinase inhibitors and newer cancer drugs notorious for their association with AF like Ibrutinib. (4) Detection of AF is not based on systematic screening but depended on either the clinical diagnosis of AF or capture of AF on an ECG, and therefore some patients who truly have AF may have been missed. In addition, given the fact that the mean follow up duration after cancer is about 5 years (35), AF that develops later, especially as a result of radiation induced fibrosis, may be missed. (5) Radiation doses were not uniformly documented and therefore further dose response could not be ascertained. Only 8% of cancer patients received radiation therapy, consequently subgroup analysis of AF risk based on site of radiation could not be performed. (6) Although, the control population in this study did not have malignancy, most of them had non-malignant hematological conditions and could have introduced a selection bias. (7) Comorbidities were based on ICD-9 and ICD-10 codes and therefore specific variables like cardiomyopathy could not be better defined as we lacked left ventricular ejection fraction data as a discrete variable in EMR.

## Conclusion

In conclusion, results from our study add to the growing evidence in the literature that AF is more prevalent in cancer patients. This association could be related to the co-morbidities of cancer patients or other mechanisms related to the cancer and/or chemoradiotherapy. In addition, our study offers new evidence that radiation therapy for cancer is associated with an increased risk for AF. Further study is needed to evaluate this association.

## Data Availability Statement

The raw data supporting the conclusions of this article will be made available by the authors, without undue reservation.

## Ethics Statement

The studies involving human participants were reviewed and approved by Louisiana State University Health Sciences Center, Shreveport IRB. Written informed consent for participation was not required for this study in accordance with the national legislation and the institutional requirements.

## Author Contributions

PDo, NA, PDh, RM, and JM: study concept and design. PDo, NA, and PDh: literature search. NA, PDh, LY, UM, DD, KH, DM, JM, RM, and PDo: acquisition, analysis, and interpretation of data. PDo, LY, PDh, NA, UM, MB, DM, and DG: drafting of the manuscript. PDo, DM, DG, JM, RM, and MB: critical revision of the manuscript for important intellectual content. JM: statistical analysis. PDo and RM: administrative, technical, or material support. PDo and RM: study supervision. All authors contributed to the article and approved the submitted version.

## Conflict of Interest

The authors declare that the research was conducted in the absence of any commercial or financial relationships that could be construed as a potential conflict of interest.

## Abbreviations

LSU: Louisiana State University
ICD: International Statistical Classification of Diseases and Related Health Problems
AF: atrial fibrillation
OR: odds ratio
ECG: electrocardiogram
EMR: electronic medical records.
